# Comparative Transcriptome Analysis of the Response to *Vibrio parahaemolyticus* and Low-Salinity Stress in the Swimming Crab *Portunus trituberculatus*

**DOI:** 10.3390/biology12121518

**Published:** 2023-12-12

**Authors:** Dongfang Sun, Jianjian Lv, Yukun Li, Jie Wu, Ping Liu, Baoquan Gao

**Affiliations:** 1 National Key Laboratory of Mariculture Biobreeding and Sustainable Goods, Yellow Sea Fisheries Research Institute, Chinese Academy of Fishery Sciences, Qingdao 266071, China; sundf@ysfri.ac.cn (D.S.); lvjj@ysfri.ac.cn (J.L.); liyukun969@163.com (Y.L.); 17806287246@163.com (J.W.); liuping@ysfri.ac.cn (P.L.); 2 Laboratory for Marine Fisheries Science and Food Production Processes, Laoshan Laboratory, Qingdao 266237, China

**Keywords:** *Portunus trituberculatus*, *Vibrio parahaemolyticus*, low salinity

## Abstract

**Simple Summary:**

In this study, we investigated the effects of pathogen infection and low-salinity stress on organismal immunity during *Portunus trituberculatus* culture by utilizing transcriptome technology. We depicted the dynamic expression process of the transcriptome under pathogenic infection and low-salinity stress. In addition, several immune genes were screened that were jointly affected by immune infection and low-salinity stress. These results contribute to our understanding of the impact of environmental factors on the immunoregulatory mechanisms of *P. trituberculatus*.

**Abstract:**

*Vibrio parahaemolyticus* is one of the main pathogenic bacteria of *Portunus trituberculatus* and causes mass mortality of *P. trituberculatus* in aquaculture. In addition, low-salinity stimulation makes *P. trituberculatus* more susceptible to *V. parahaemolyticus* infections. In order to elucidate the molecular mechanism of resistance to *V. parahaemolyticus* in *P. trituberculatus*, comparative transcriptomic analysis of blood cells stimulated by low salinity and *V. parahaemolyticus* was carried out in this study. Transcriptome sequencing of low-salinity stress and pathogen infection at different time points was completed using Illumina sequencing technology. A total of 5827, 6432, 5362 and 1784 differentially expressed genes (DEGs) involved in pathways related to ion transport and immunoregulation were found under low-salinity stress at 12, 24, 48 and 72 h compared with the control at 0 h. In contrast, 4854, 4814, 5535 and 6051 DEGs, which were significantly enriched in Toll and IMD signaling pathways, were found at 12, 24, 48 and 72 h compared with the control at 0 h under *V. parahaemolyticus* infection. Among them, 952 DEGs were shared in the two treatment groups, which were mainly involved in apoptosis and Hippo signaling pathway. Cluster analysis screened 103 genes that were differentially expressed in two factors that were negatively correlated, including immunoglobulin, leukocyte receptor cluster family, scavenger receptor, macroglobulin and other innate-immune-related genes. These results provide data support for the analysis of the mechanisms of immunity to *V. parahaemolyticus* under low-salinity stress in *P. trituberculatus* and help to elucidate the molecular mechanisms by which environmental factors affect immunity.

## 1. Introduction

The swimming crab *Portunus trituberculatus* belongs to Crustacea, Decapoda, Portunidae, and is widely distributed in the coastal waters of China, Korea and Japan. The swimming crab possesses the characteristics of fast growth and strong adaptability. In China, the annual aquaculture production of swimming crab, which has become another important economic crustacean following shrimps and blue crabs, reaches 120,000 tons. However, with the increasing scale of aquaculture, intensive aquaculture results in the frequent occurrence of diseases [1,2], among which *Vibrio parahaemolyticus* is a major pathogen causing diseases in *P. trituberculatus* [3,4]. This pathogen causes hepatopancreatic tissue damage and slow growth in swimming crabs, thus generating huge economic losses in the aquaculture industry. However, the outbreak of disease is commonly associated with alterations in the environment. Environmental stresses induce decreased immunity and increased sensitivity to pathogenic infections in crustaceans [5]. For instance, an increase or decrease in salinity can lead to a decrease in the immunity of tiger shrimp [6]. In addition, changes in temperature, dissolved oxygen, ammonia nitrogen and biological contaminants also affect the immune response [5]. Immunological studies on *V. parahaemolyticus* in crustaceans have been reported; e.g., immune genes showed corresponding expression patterns in response to infection [7,8]. However, fewer reports are available on the mechanism of immune response in swimming crabs under environmental stress.

Innate immunity is an important way in which crustaceans resist pathogen invasion, and immune-related genes perform vital roles in this process. Transcriptome sequencing technology is widely applied to mine genes related to traits such as immunity, body color and sex in aquatic animals [9,10,11,12,13,14], and its utilization has been reported in species such as fish, shrimp and shellfish to explore the expression patterns of genes related to growth, development and sex [9,12,13]. More studies have reported utilizing comparative transcriptomics to mine crustacean immune-related genes, mainly focusing on economic crustaceans, including *Litopenaeus vannamei*, *Eriocheir sinensis* and *Scylla paramamosain* [15,16,17,18,19,20,21], which provided an essential foundation for analyzing the immune mechanisms of crustaceans.

In this study, blood cells at 0, 12, 24, 48 and 72 h post low-salinity stress and *V. parahaemolyticus* treatment were analyzed for the comparative analysis of dynamic transcriptome sequencing. The results are of great significance in analyzing the immune mechanism of *P. trituberculatus*, as well as contributing to the understanding of the molecular mechanisms of the effects of environmental stress on the immune system.

## 2. Materials and Methods

### 2.1. Animals

Swimming crabs (80 days, 25.6 ± 3.2 g) were collected from a full-sib family line from a local aquaculture farm in Weifang, Shandong Province, China. Prior to commencing the formal experiment, the swimming crabs underwent a one-week acclimation period. During this period, the water temperature was consistently maintained at 24 °C, oxygen was continually introduced into the aquatic environment, and miscellaneous fish were periodically fed according to the previous study [22]. The assay was divided into a low-salinity group (Ls, salinity 11‰) and a challenge group (Vp, *V. parahaemolyticus* concentration of 10^5^ CFU/mL) with 60 swimming crabs in each group. In the challenge group, 100 μL of *V. parahaemolyticus* was injected into the basement membrane of the first joint at the base of the swimming foot. The *V. parahaemolyticus* used in this study was obtained from the Research Laboratory of Disease Control and Molecular Pathology of Cultured Organisms, Yellow Sea Fisheries Research Institute. At the same time, we obtained authorization from the Yellow Sea Fisheries Research Institute to use the pathogen for infection experiments. The feeding conditions during the experiment were consistent with those during the temporary feeding period. The blood cells were taken from three crabs randomly selected from each group at 0 h, 12 h, 24 h, 48 h and 72 h, and 0 h served as the control group. Swimming crabs were treated with hypothermia to induce cold shock, and sampling of blood cells was performed according to the previous procedure [22]. Three samples were taken at each time point, and each sample was generated from a mixture of blood cells from five crabs.

### 2.2. Library Preparation and RNA-Seq

The RNA was isolated from the blood cells by utilizing the TRIzol method according to the manufacturer’s instructions. RNA integrity was assessed using the RNA Nano 6000 Assay Kit of the Bioanalyzer 2100 system (Agilent Technologies, Santa Clara, CA, USA). A total amount of 1 μg RNA per sample was used as input material for the RNA sample preparations. Briefly, mRNA was purified from total RNA using poly-T oligo-attached magnetic beads. Fragmentation was carried out using divalent cations under elevated temperature in First Strand Synthesis Reaction Buffer(5×). First-strand cDNA was synthesized using random hexamer primer and M-MuLV Reverse Transcriptase (RNase H-). Second-strand cDNA synthesis was subsequently performed using DNA Polymerase I and RNase H. Remaining overhangs were converted into blunt ends via exonuclease/polymerase activities. After adenylation of 3′ ends of DNA fragments, adaptors with a hairpin loop structure were ligated to prepare for hybridization. In order to select cDNA fragments preferentially 370~420 bp in length, the library fragments were purified with the AMPure XP system (Beckman Coulter, Beverly, CA, USA). Then, PCR was performed with Phusion High-Fidelity DNA polymerase, Universal PCR primers and Index (X) Primer. Finally, PCR products were purified (AMPure XP system) and library quality was assessed on the Agilent Bioanalyzer 2100 system. The library preparations were sequenced on an Illumina Novaseq platform, and 150 bp paired-end reads were generated.

### 2.3. Data Analysis

The raw data were processed for quality control using Fastp software (v0.23.1) to obtain cleandata. All the downstream analyses were based on the clean data with high quality. The reference genome (GCA_020740555.1) was indexed using Hisat2 (v2.0.5), and clean reads were aligned to the reference genome by utilizing Hisat2 with default parameters. FeatureCounts (v1.5.0) was used to count the reads numbers mapped to each gene. And then FPKM (fragments per kilobase of exon model per million mapped fragments) of each gene was calculated based on the length of the gene and reads count mapped to the gene. Differentially expressed genes (DEGs) analysis of two groups was performed using the DESeq2 (v1.20.0) with default parameters. DESeq2 provides statistical routines for determining differential expression in digital gene expression data using a model based on the negative binomial distribution. The resulting *p*-values were adjusted using Benjamini and Hochberg’s approach for controlling the false discovery rate. Only genes with *p*-value < 0.05 calibrated by false discovery rate (FDR) and absolute value of log2FoldChange > 0 were considered as DEGs.

### 2.4. GO and KEGG Enrichment Analysis of DEGs

GO functional enrichment analysis of differential gene sets was performed using clusterProfile software (v4.0), with padj < 0.05 as the threshold for being significantly enriched. KEGG is a database resource for understanding high-level functions and utilities of the biological system, such as the cell, the organism and the ecosystem, from molecular-level information, especially large-scale molecular datasets generated by genome sequencing and other high-through put experimental technologies (http://www.genome.jp/kegg/, accessed on 1 April 2023). We used clusterProfiler R package to test the statistical enrichment of differential expression genes in KEGG pathways.

### 2.5. Quantitative Real-Time PCR Validation

The RNA was isolated from the blood cells by utilizing the Trizol method according to the manufacturer’s instruction. Blood cells from five swimming crabs were mixed into one sample, and three biological replicates were used for each time point. cDNA was reverse-transcribed from 1 μg of RNA using the HiScript II Q RT SuperMix for RT-qPCR (+gDNA wiper) kit (Vazyme, Nanjing, China) according to the instructions. RT-qPCR was performed following a 5-fold dilution of the cDNA. The volume of the qPCR reaction system was 20 μL, containing 10 μL of 2 × SYBR Master Mix (Takara, Osaka, Japan), 0.4 μL of primer (Table S1) and 0.2 μL of ROX Reference Dye II, 2 μL of cDNA template and RNase-free water. qPCR was carried out in a 7500 Fast system (ThermoFisher, Waltham, MA, USA) according to previous research [22]. The β-actin gene was used as an endogenous control, and relative expression levels were determined utilizing the 2^−ΔΔCt^ method [23]. The RT-qPCR data were presented as the mean ± S.D.

## 3. Result

### 3.1. Information Obtained from the Sequencing Data

In this study, in order to study the interaction between environment and immunity, Illumina Hiseq sequencing technology was used to sequence the transcriptome of blood cell samples from 0 (control), 12, 24, 48 and 72 h under low salinity and pathogen challenge. The mortality rates at 12, 24, 48 and 72 h of pathogenic infection were 18.2%, 9.4%, 3.22% and 1.02%, respectively. The sequencing results show that a total of 174.85G of raw data was obtained, with an average coverage depth of 7.83. The QC results show that the average values of Q20 and Q30 were above 97% and 92%, respectively, with an average mapping rate of 86.46% (Table S2). Ten differentially expressed genes were randomly selected for data validation, and the results show that the RT-qPCR results are basically consistent with the FPKM trend, indicating the accuracy of the sequencing results (Figure S1). All raw data were uploaded to the NCBI (National Center for Biotechnology Information, accession number: PRJNA1039533 and PRJNA1039325).

### 3.2. Dynamic Expression of the Transcriptome under Low Salinity and Pathogenic Challenge

**Figure 1:**
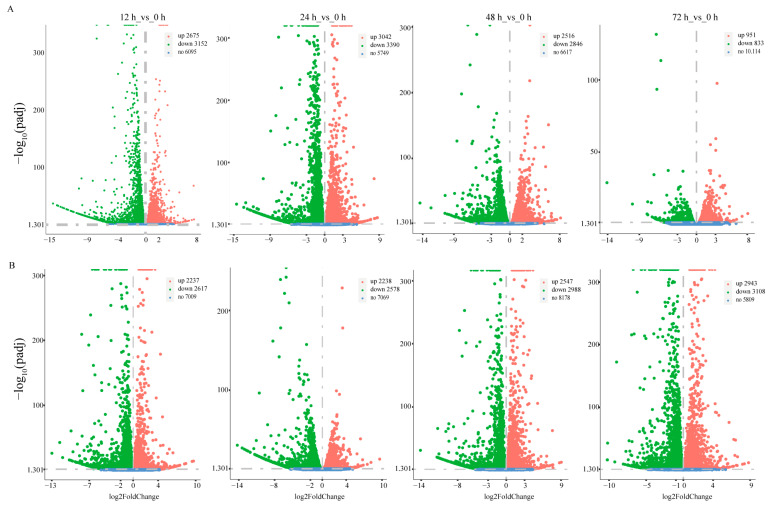
The number of DEGs at different time points post *V. parahaemolyticus* (**A**) and low-salinity (**B**) stress. “up” indicates the number of DEGs that were upregulated, “down” indicates the number of DEGs that were downregulated, and “no” indicates the number of genes that were not significantly different.

**Figure 2:**
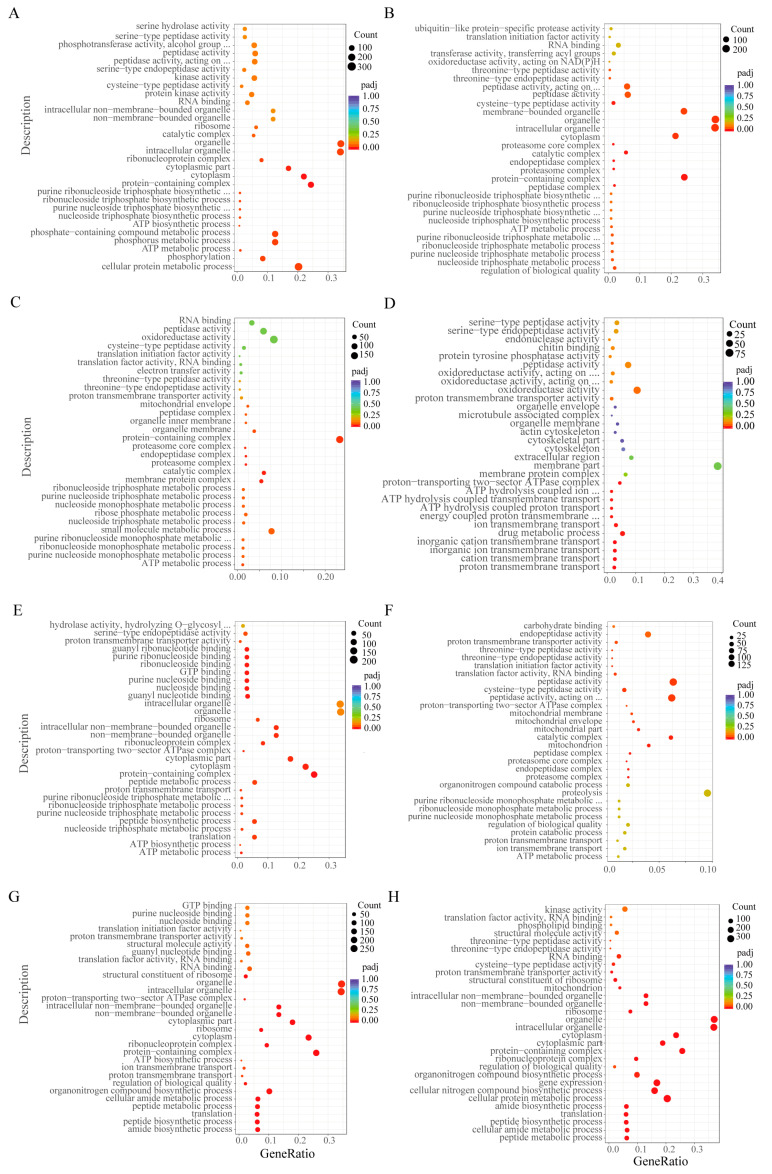
GO enrichment analysis of DEGs under low-salinity and *V. parahaemolyticus* stress. (**A**–**D**) GO enrichment analysis of DEGs at 12, 24, 48 and 72 h under low-salinity stress compared with 0 h. (**E**–**H**) GO enrichment analysis of DEGs at 12, 24, 48 and 72 h under *V. parahaemolyticus* stress compared with 0 h.

**Figure 3:**
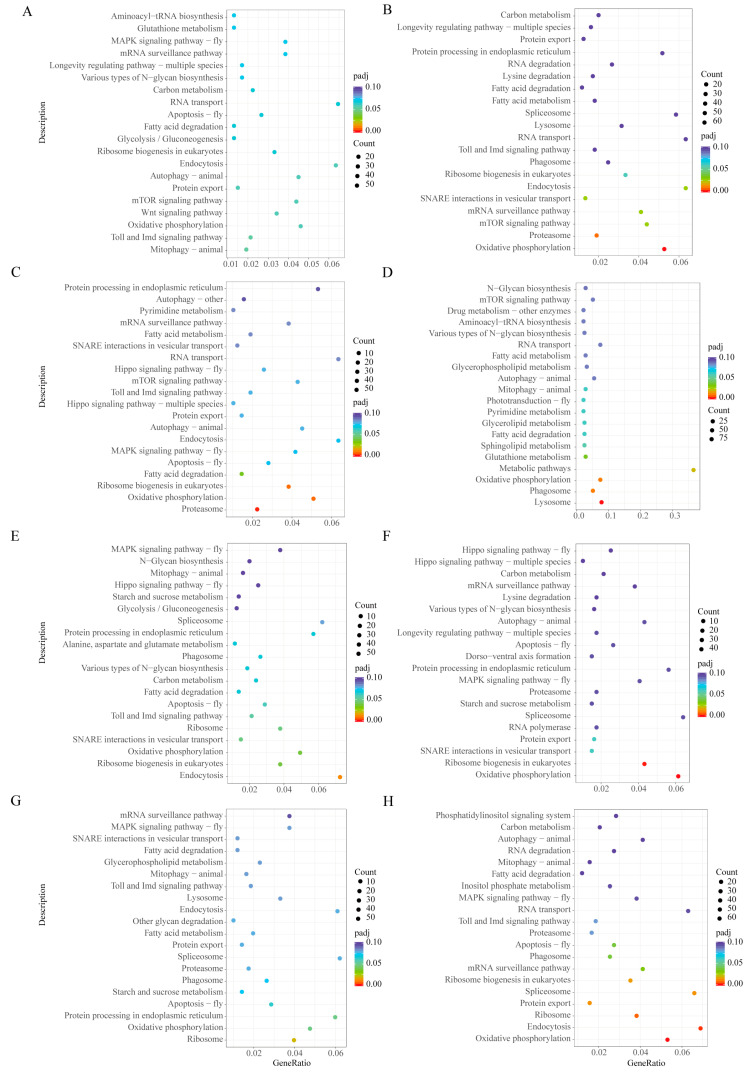
KEGG annotation of DEGs under low salinity and *V. parahaemolyticus*. (**A**–**D**) KEGG annotation of DEGs at 12, 24, 48 and 72 h under low-salinity stress compared with 0 h. (**E**–**H**) KEGG annotation of DEGs at 12, 24, 48 and 72 h under *V. parahaemolyticus* stress compared with 0 h.

A total of 5827, 6432, 5362 and 1784 DEGs were found under low-salinity stress at 12, 24, 48 and 72 h compared with the control at 0 h. The number of DEGs tended to increase and then decrease as the duration of stress increased (Figure 1A). In contrast, 4854, 4814, 5535 and 6051 DEGs were found at 12, 24, 48 and 72 h compared with the control at 0 h. The number of DEGs increased gradually with the duration of pathogen stress (Figure 1B). Under low-salinity stress, DEGs were mainly enriched in biological processes related to energy metabolism and ion transport, such as the ATP metabolic process and ion transmembrane transport. These differential genes were mainly involved in apoptosis and proteasome pathways (Figure 2A–D). Surprisingly, they were also enriched for the immune-related pathways Toll and Imd signaling pathways (Figure 3A–D and Figure S2). Differential genes under pathogen stress were mainly related to peptidase synthesis and activity, while differential genes were significantly enriched in immune-related signaling pathways, such as Toll and IMD signaling pathways (Figure 2E–H and Figure 3E–H).

### 3.3. Expression Pattern Clustering of DEGs

**Figure 4:**
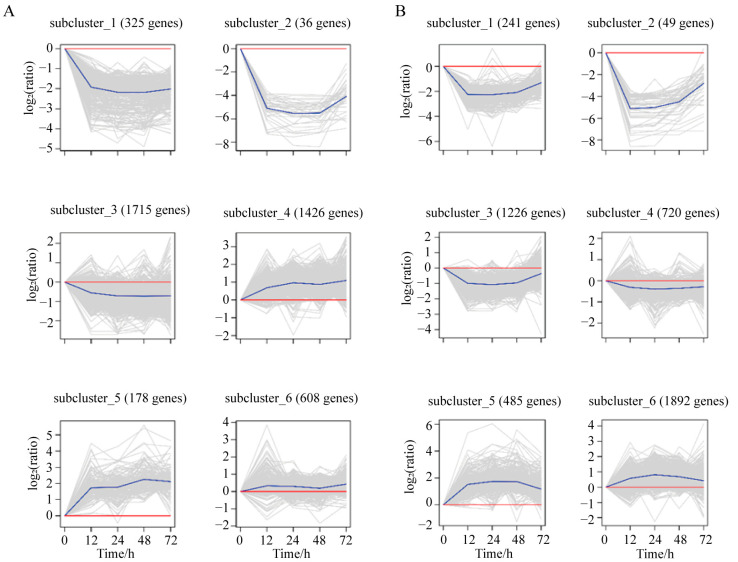
Cluster analysis of differential genes. (**A**) DEG clustering analysis of *V. parahemolyticus* challenge group. (**B**) DEG clustering analysis of low-salinity group. Each subplot shows the expression levels of genes in a subcluster, where the *X*-axis represents the stress treatment time and the *Y*-axis represents the relative expression levels of DEGs. Each gray line is the relative expression of a specific gene at different stress times, while the blue line represents the average relative expression of all genes in the subcluster, and the red line represents the baseline.

A cluster analysis was performed on the DEGs in the infection and low-salinity groups, and the results reveal that each of the two groups of DEGs was clustered into six categories (Figure 4). In the infection group, subcluster 3 contained the largest number of DEGs, with an initial trend of downregulated expression (Figure 4A). Subcluster 6 was the largest cluster of DEGs in the low-salinity group, with an overall expression trend of upregulation (Figure 4B). According to the results of cluster analysis, 2212 genes showed an overall upregulation trend post infection (subclusters_4, 5 and 6), and 2076 genes showed an overall downregulation trend post infection (subclustesr_1, 2 and 3); there were 2377 genes that showed an overall upward regulation trend after salinity stress (subclusters_5 and 6), and 2236 genes that showed an overall downregulation trend (subclusters_1, 2, 3 and 4).

### 3.4. Screening of Reverse DEGs after Exposure to the Two Kinds of Stress

**Figure 5:**
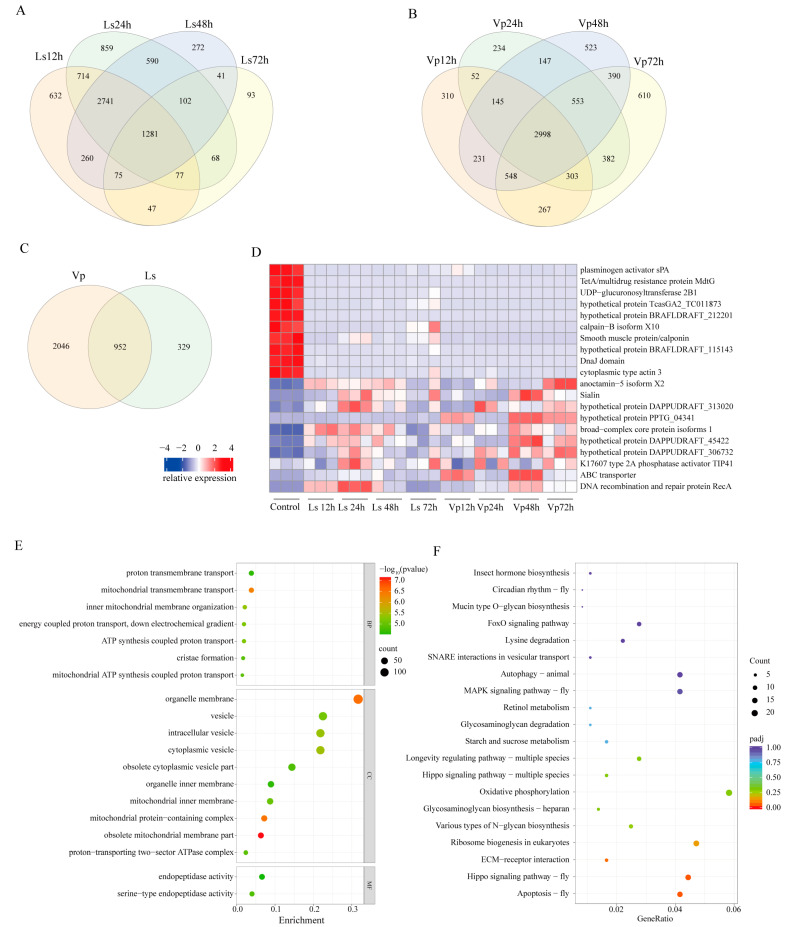
Co-analysis of DEGs under low salinity and pathogenic infections. (**A**) Number of shared DEGs at four time points under low-salinity stress. (**B**) Number of shared DEGs at four time points under pathogen infection. (**C**) Number of shared differential genes under low-salinity and pathogen stress. (**D**) Expression pattern of top 10 DEGs under low-salinity and pathogen stress. (**E**) GO enrichment analysis of shared DEGs. (**F**) KEGG enrichment analysis of shared DEGs.

A total of 1281 genes were found to be consistently differentially expressed throughout the low-salinity treatment (Figure 5A). Surprisingly, these genes were mainly enriched in immune-related signaling pathways (Figure S3A). In contrast, a total of 2998 genes were consistently differentially expressed under pathogen stress (Figure 5B), and they were mainly enriched in immune-related signaling pathways (Figure S3B). Looking at the intersection of the DEGs under the treatment of two stress factors, we screened 952 shared genes (Figure 5C). These genes were mainly involved in biological processes related to osmotic pressure regulation, such as proton transmembrane transport (Figure 5D). Furthermore, they were involved in signaling pathways related to immunity and osmotic pressure regulation, such as apoptosis (Figure 5E).

Based on the results of cluster analysis, DEGs with opposite trends in expression patterns after pathogen infection and low-salinity stress were screened out. Among them, 44 DEGs were upregulated post pathogen infection and downregulated following low-salinity stress (vp_up and ls_down). This was mainly due to the significant upregulation of expression by pathogen infection after 72 h, including for immunoglobulin genes, leukocyte receptor cluster family genes, scavenger receptors, macroglobulins and other genes related to innate immunity. In addition, 59 DEGs were downregulated following pathogen infection and upregulated following low-salinity stress (vp_down and ls_up). This was mainly due to the significant upregulation of expression under low-salinity stress, including chloride ion channels, calcium channels and other genes. The results of KEGG enrichment analysis show that the vp_up and ls_down group was mainly enriched in the Wnt signaling pathway, and the vp_down and ls_up group was not enriched in a significant pathway (Table S3).

## 4. Discussion

Immune protection in crustaceans is mainly accomplished by blood cells, which play an important role in the innate immune response of crustaceans. Blood cells are distributed throughout the body via blood circulation and play a role in the host’s immune response in terms of recognition, phagocytosis, melanogenesis, cytotoxicity and cellular communication as well as immune defenses such as phagocytosis, nodules and cysts [24]. *V. parahaemolyticus* is a major pathogen in the culture of *P. trituberculatus*, which can cause severe pathological damage to tissues, slow growth and difficulty in shelling [25]. Salinity is an environmental factor that cannot be ignored in aquaculture, especially in outdoor pond culture mode. During the rainy season, the reduced salinity of farmed seawater puts *P. trituberculatus* in a low-salinity stress environment, which increases its susceptibility to *V. parahaemolyticus* infection. In this study, the transcriptome dynamics analysis of blood cells posts *V. parahaemolyticus* and salinity stress was carried out. A large number of post-stress DEGs were identified, and correlation analysis of post-stress differential genes between the two factors helped us to analyze the mechanisms of immunity and stress resistance in this species.

*P. trituberculatus* is a hyperosmotic-regulated species [26]. In the early stage of low-salinity stress, a large number of differential genes were involved in osmotic pressure regulation in the swimming crab, involving a variety of ionic regulation and capacity metabolic pathways. At the later stage of stress, the osmotic pressure in the swimming crab maintained a relative equilibrium with the environmental osmotic pressure, and the number of differential genes decreased. Meanwhile, we found that the differential genes were significantly enriched in signaling pathways related to immunity, which is consistent with studies in other crustaceans and suggests that environmental factors may induce immune responses [27,28]. *V. parahaemolyticus* is a major pathogen of crustaceans. In this study, a large number of individuals died 12 h post pathogen injection, followed by a gradual decrease in mortality. As the duration of pathogen challenge increased, more genes responded to the infection stimulus and participated in the immune response. Many crustaceans have shown that the Toll, IMD and Jak-STAT signaling pathways are involved in the immune regulation of *V. parahaemolyticus* infection [21,29,30]. In this study, the differential genes were significantly enriched in the Toll and IMD signaling pathways, in line with the findings in other crustaceans.

In this study, the expression of differential genes during salinity and pathogen treatments provided us with the dynamic expression patterns of *P. trituberculatus* in response to different stresses and clarified the transcriptional regulatory mechanisms of *P. trituberculatus* in response to salinity and pathogen stresses. The DEGs following pathogenic infection were associated with immunity, such as chitin and lectin [31,32], suggesting that they participate in the resistance of *P. trituberculatus* to invasion by parahaemolyticus disease. In contrast to the gill transcriptome results [33], fewer specific pathways were enriched for DEGs associated with salinity acclimation in hemocytes after low-salinity stress. Therefore, it was hypothesized that hemocytes do not possess a major function in salinity adaptation and that these differential genes may be aberrantly expressed only in low-salinity stimulation.

Notably, the number of DEGs shared by the two groups was 952, and they were mainly enriched in apoptosis and the Hippo signaling pathway. Apoptosis plays a necessary role in the removal of unwanted or abnormal cells in multicellular organisms. The addition of AP to feed reduces ROS accumulation and apoptosis in shrimp, thereby protecting the shrimp from *V. albicans infection* [34]. The Hippo signaling pathway is an evolutionarily conserved pathway that controls organ size by regulating cell proliferation, apoptosis and stem cell self-renewal [35]. These pathways have been reported to be associated with innate immunity of crustaceans [36,37], suggesting that these pathways were involved in the resistance to *V. parahaemolyticus* and were affected by low-salinity stress in *P. trituberculatus*.

Under low-salt stress, crustaceans enhance their immunity by increasing lipid metabolism, glucose metabolism, detoxification and osmotic sensitivity regulation, suggesting that lower immune responses under low-salinity stress indicate a state of immunosuppression and increased disease susceptibility [5]. In the crustacean culture, a large number of deaths were induced after heavy rainfall. *P. trituberculatus* under low-salinity stress was more sensitive to *V. parahaemolyticus* infection, and it was hypothesized that low-salinity stress may lead to reduced immunity by affecting immunity or altering the expression patterns of relevant genes. In this study, we screened a total of 103 DEGs, including 44 DEGs that were upregulated after pathogenic infection and downregulated by low-salinity stress and 59 DEGs that were downregulated after pathogenic infection and upregulated after low-salinity stress. These genes included immunoglobulin genes, leukocyte receptor cluster families, scavenger receptors, macroglobulins and other genes related to innate immunity and stress resistance [38,39,40,41,42,43,44,45], which may play important roles in the reduction in immunity in *P. trituberculatus* under low-salinity stress.

## 5. Conclusions

In this study, we analyzed the dynamic expression patterns of differential genes during low-salinity and pathogen stresses. The differential genes after pathogen stress were mainly enriched in apoptosis, ribosome biogenesis in eukaryotes, Hippo signaling and other pathways. The number of DEGs shared by the two groups was 952, suggesting that a large number of genes may function in both disease resistance and stress adaptation. Our results provide data support for analyzing the mechanism of immunity to *V. parahaemolyticus* in *P. trituberculatus* and help to elucidate the molecular mechanisms by which salinity affects immunity.

## Data Availability

Data are contained within the article and supplementary materials.

## Supplementary Materials

The following supporting information can be downloaded at: https://www.mdpi.com/article/10.3390/biology12121518/s1. Figure S1: qPCR validation of transcriptome data. The RT-qPCR data were presented as the mean ± S.D. Figure S2: qPCR validation of genes in the Toll and IMD signaling pathways. TLR1, Toll-like receptors 1; TLR2, Toll-like receptors 2; SR, scavenger receptor; Mapkkk4, mitogen-activated protein kinase kinase kinase 4; Mapkkk7, mitogen-activated protein kinase kinase kinase 7; Mapkk6, mitogen-activated protein kinase kinase 6. Different lowercase letters indicate significant differences (*p* < 0.05) in RNA-seq; different uppercase letters indicate significant differences (*p* < 0.05) in qPCR. Figure S3: KEGG annotation of DEGs under low salinity and *V. parahaemolyticus*. (A) KEGG annotation of shared DEGs under low-salinity stress. (B) KEGG annotation of shared DEGs under pathogen stress. Table S1: primer sequences for qPCR. Table S2: statistics of the transcriptome sequencing from blood samples of *P. trituberculatus*. Table S3: KEGG of DEGs with opposite trends in expression patterns post pathogen infection and low-salinity stress.
