# Supplementary material for: Comparative Transcriptome Analysis of the Response to Vibrio parahaemolyticus and Low-Salinity Stress in the Swimming Crab Portunus trituberculatus
Source: Biology (Basel). 2023 Dec 12;12(12):1518. doi: 10.3390/biology12121518 (PMC10741082; doi:10.3390/biology12121518)
Supplement: Supplementary file 1 [file biology-12-01518-s001.zip › biology-2680014-supplementary.pdf]

biology MDPI

Supplementary Materials

**Table S1.** Primer sequences for qPCR.

| Gene name | Forward primer（5'-3'） | Reverse primer（5'-3'） |
|---|---|---|
| Clpro | CACCCACGCAGATTGGAACT | TGACGATGGTGCCCACAGAC |
| Durt1 | TACCACGCCGCCATACCCAA | GCAGGCTGACTGATACTGAAGGC |
| Chymotrypsin | ACATCAGGTTGGAAGAAGCGTC | GTGTTGAATGTGACGGGGCT |
| Hemocyanin | TGGTGTGGCTCGCATCAGA | GCACCCACGACATCAATACCAT |
| Vmo1 | AAGAGGGGTGACTGGCAAGG | TCCACTCCAAAGTCATCGCC |
| Hemocyanin A | GAAGTCAAGGCTGGAACGCA | CATCATTCACAGCCTCGTCAGTC |
| Nodulin-75 | CACCTACACTCCTTCCCTGGCA | TTAGGCAGCGACGAACTTGAA |
| CD97 | CCTACAAGAACGGTGGAGCG | TGGGGAAAAGAATGTTGGGA |
| Durt2 | TGACATCTTACCTGAACTGCCAAA | AGGCGGTAGTTATCCATCCCA |
| Iscw | AAGATGGAATAATAGTCCTTACGGC | AGGCGGGAGCAAATCACTG |
| TLR1 | GCTTGGAGTACACCCAGAA | TTAACAAGTCCGTCCAGGTG |
| TLR2 | GATCCCAGCAGAGTCTGAGC | CAAGATCTGGTGGATGGGGC |
| SR | GCACTACTGTCCCTGGCAAT | GCCCTCCACACCATTGAGAA |
| Mapkkk4 | TTCAGTCTGTGTGAGGAGCC | GGTGGGTGGATGGGTATCA |
| Mapkkk7 | GTGGCAGTGAAGAAGGTAGAAA | GCAAACTCAATGACCAGGCA |
| Mapkk6 | CGCTCAGATGTGTGGTCACT | GAAAGTTCCAGGAGGGAGGC |

**Table S2.** Statistics of the transcriptome sequencing from blood samples of *P. trituberculatus*.

| Sample | Raw_reads | Clean_reads | Q20 (%) | Q30 (%) | Mapped to genome (%) | Coverage depth |
|---|---|---|---|---|---|---|
| 0h_1 | 45,218,876 | 44,011,870 | 97.46 | 93.1 | 88.54% | 7.97 |
| 0h_2 | 37,195,138 | 36,084,876 | 97.69 | 93.63 | 88.79% | 8.27 |
| 0h_3 | 45,525,094 | 44,600,106 | 97.89 | 94.35 | 88.59% | 8.01 |
| Ls_12h_1 | 46,859,006 | 45,828,352 | 98.17 | 94.92 | 87.23% | 8.24 |
| Ls_12h_2 | 45279,026 | 44,062,912 | 98.06 | 94.5 | 87.70% | 7.93 |
| Ls_12h_3 | 42,583,858 | 41,359,654 | 98.08 | 94.65 | 86.33% | 7.43 |
| Ls_24h_1 | 47,453,074 | 46,231,682 | 97.92 | 94.35 | 84.48% | 8.31 |
| Ls_24h_2 | 45,837,770 | 44,449,438 | 98.01 | 94.57 | 84.96% | 8.00 |
| Ls_24h_3 | 44,245,524 | 43,263,644 | 97.93 | 94.29 | 85.09% | 7.78 |
| Ls_48h_1 | 46,152,442 | 45,084,506 | 98.13 | 94.79 | 85.96% | 8.11 |
| Ls_48h_2 | 49,249,936 | 48,575,640 | 97.65 | 93.72 | 87.32% | 8.74 |
| Ls_48h_3 | 45,747,746 | 45,034,808 | 97.61 | 93.64 | 87.06% | 8.11 |
| Ls_72h_1 | 45,811,612 | 44,415,824 | 97.65 | 93.74 | 86.42% | 7.99 |
| Ls_72h_2 | 41,454,254 | 40,265,614 | 97.8 | 94.12 | 86.06% | 7.24 |
| Ls_72h_3 | 45,655,424 | 44,919,668 | 97.54 | 93.63 | 85.94% | 8.08 |
| Vp_12h_1 | 43,997,912 | 43,196,466 | 97.35 | 93.14 | 86.61% | 7.77 |
| Vp_12h_2 | 38,543,930 | 37,381,920 | 97.52 | 93.57 | 86.82% | 6.73 |
| Vp_12h_3 | 43,404,780 | 42,550,502 | 97.59 | 93.71 | 86.88% | 7.65 |
| Vp_24h_1 | 44,069,140 | 43,047,190 | 97.56 | 93.43 | 85.74% | 7.75 |

| | | | | | | |
|---|---|---|---|---|---|---|
| Vp_24h_2 | 38,601,836 | 37,812,006 | 97.24 | 92.7 | 85.78% | 6.80 |
| Vp_24h_3 | 42,987,796 | 41,768,994 | 97.64 | 93.8 | 87.00% | 7.52 |
| Vp_48h_1 | 46,401,016 | 45,466,782 | 97.7 | 93.83 | 86.12% | 8.18 |
| Vp_48h_2 | 42,439,408 | 41,578,678 | 97.74 | 93.98 | 86.02% | 7.48 |
| Vp_48h_3 | 47,674,034 | 46,769,312 | 97.68 | 93.88 | 85.73% | 8.42 |
| Vp_72h_1 | 46,008,204 | 45,190,418 | 97.43 | 93.35 | 85.65% | 8.13 |
| Vp_72h_2 | 43,930,664 | 43,097,034 | 97.42 | 93.3 | 85.56% | 7.75 |
| Vp_72h_3 | 40,381,368 | 39,615,108 | 97.55 | 93.58 | 86.07% | 7.12 |

**Table S3.** KEGG of DEGs with opposite trends in expression patterns post pathogen infection and low salinity stress.

| | KEGG ID | Description | BgRatio | padj | Gene name | Count |
|---|---|---|---|---|---|---|
| Vp_up & ls_down | dme04310 | Wnt signaling pathway | 64/2203 | 0.001662 | receptor tyrosine kinase | 2 |
| | dme04150 | mTOR signaling pathway | 73/2203 | 0.06519 | Frizzled-4 | 1 |
| Vp_down & ls_up | dme00760 | Nicotinate and nicotinamide metabolism | 16/2203 | 0.053146 | Frizzled-4 | 1 |
| | dme00260 | Glycine, serine and threonine metabolism | 25/2203 | 0.053146 | Cytosolic purine 5'-nucleotidase | 1 |
| | dme00513 | Various types of N-glycan biosynthesis | 25/2203 | 0.053146 | sarcosine dehydrogenase | 1 |
| | dme00510 | N-Glycan biosynthesis | 31/2203 | 0.053146 | alpha-1,3-mannosyl-glycoprotein 4-beta-N-acetylglucosaminyltransferase C-like | 1 |
| | dme00240 | Pyrimidine metabolism | 33/2203 | 0.053146 | alpha-1,3-mannosyl-glycoprotein 4-beta-N-acetylglucosaminyltransferase C-like | 1 |
| | dme00230 | Purine metabolism | 72/2203 | 0.09492 | Cytosolic purine 5'-nucleotidase | 1 |

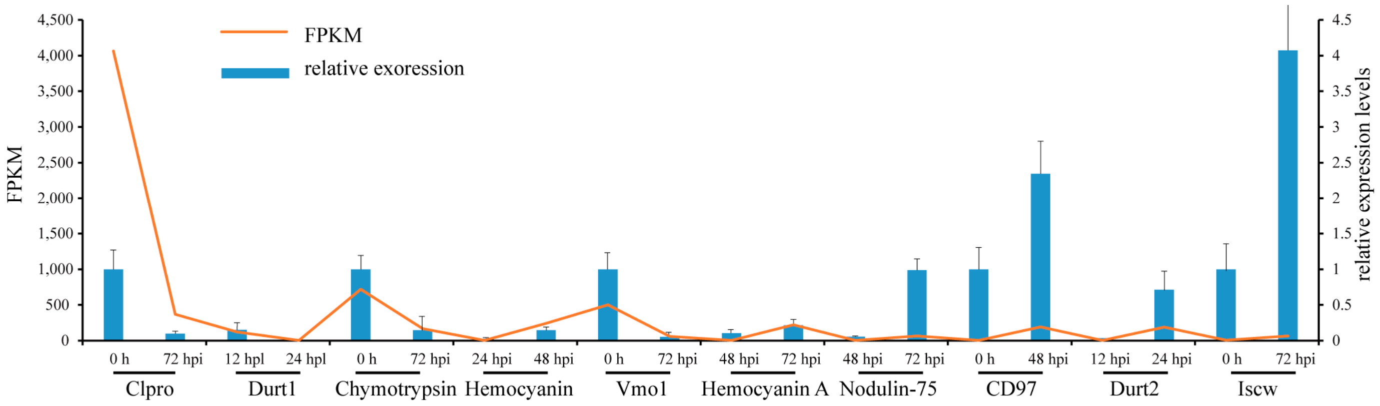


**Figure S1.** qPCR validation of transcriptome data. The RT-qPCR data was presented as the mean ± S.D.

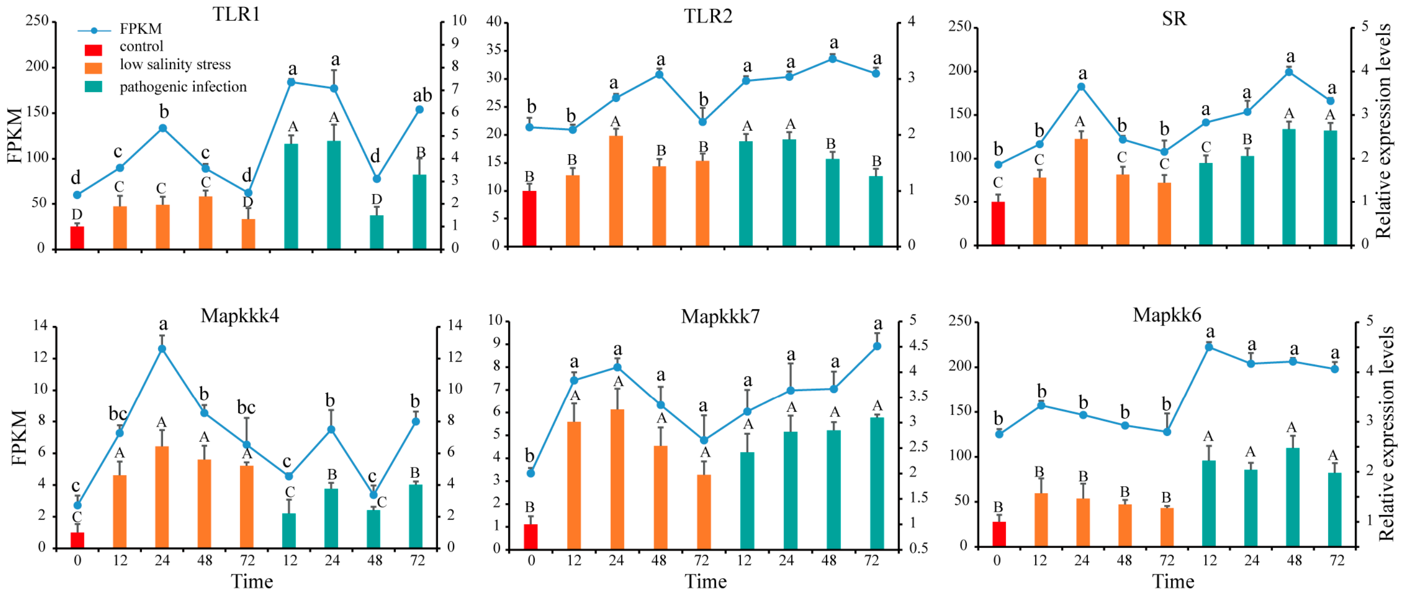


**Figure S2.** qPCR validation of genes in the Toll and IMD signaling pathways. TLR1, Toll-like receptors 1; TLR2, Toll-like receptors 2; SR, scavenger receptor; Mapkkk4, mitogen-activated protein kinase kinase kinase 4; Mapkkk7, mitogen-activated protein kinase kinase kinase 7; Mapkk6, mitogen-activated protein kinase kinase 6. Different lowercase letters indicate significant differences (*P*<0.05) in RNA-seq; different uppercase letters indicate significant differences (*P*<0.05) in qPCR.

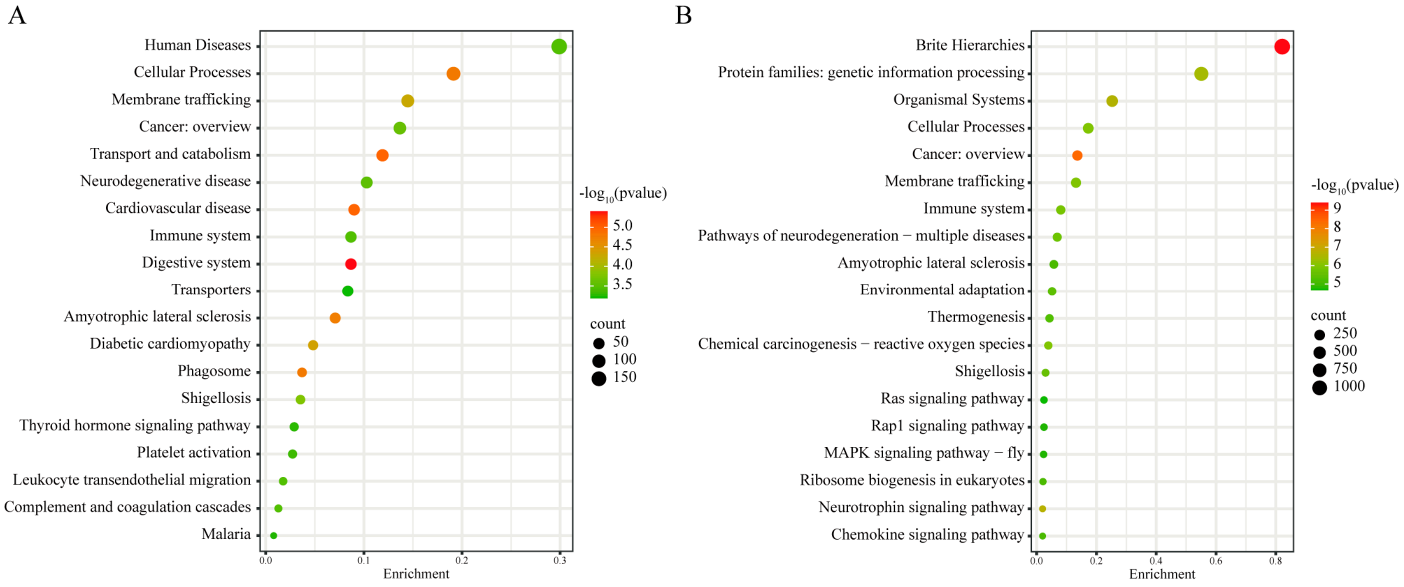


**Figure S3.** KEGG annotation of DEGs under low salinity and *V. parahaemolyticus*. (A) KEGG annotation of shared DEGs under low salinity stress. (B) KEGG annotation of shared DEGs under pathogen stress.
